# The Evolutionary History and Diverse Physiological Roles of the Grapevine Calcium-Dependent Protein Kinase Gene Family

**DOI:** 10.1371/journal.pone.0080818

**Published:** 2013-12-06

**Authors:** Fei Chen, Marianna Fasoli, Giovanni Battista Tornielli, Silvia Dal Santo, Mario Pezzotti, Liangsheng Zhang, Bin Cai, Zong-Ming Cheng

**Affiliations:** 1 Fruit Crop Systems Biology Laboratory, College of Horticulture, Nanjing Agricultural University, Nanjing, China; 2 Dipartimento di Biotecnologie, Università degli Studi di Verona, Verona, Italy; 3 Department of Biology, Huck Institutes of the Life Sciences, Pennsylvania State University, University Park, Pennsylvania, United States of America; 4 Department of Plant Sciences, University of Tennessee, Knoxville, Tennessee, United States of America; CNRS/University Lyon 1, France

## Abstract

Calcium-dependent protein kinases (CDPKs) are molecular switches that bind Ca^2+^, ATP, and protein substrates, acting as sensor relays and responders that convert Ca^2+^ signals, created by developmental processes and environmental stresses, into phosphorylation events. The precise functions of the CDPKs in grapevine (*Vitis vinifera*) are largely unknown. We therefore investigated the phylogenetic relationships and expression profiles of the 17 CDPK genes identified in the 12x grapevine genome sequence, resolving them into four subfamilies based on phylogenetic tree topology and gene structures. The origins of the CDPKs during grapevine evolution were characterized, involving 13 expansion events. Transcriptomic analysis using 54 tissues and developmental stages revealed three types of CDPK gene expression profiles: constitutive (housekeeping CDPKs), partitioned functions, and prevalent in pollen/stamen. We identified two duplicated CDPK genes that had evolved from housekeeping to pollen-prevalent functions and whose origin correlated with that of seed plants, suggesting neofunctionalization with an important role in pollen development and also potential value in the breeding of seedless varieties. We also found that CDPKs were involved in three abiotic stress signaling pathways and could therefore be used to investigate the crosstalk between stress responses.

## Introduction

Protein kinases are molecular switches and signal responders that regulate plant growth and development, as well as biotic and abiotic stress responses [1]. There are nearly 1000 protein kinases in *Arabidopsis thaliana*, accounting for ∼4% of the proteome [1]. These include 34 calcium-dependent protein kinases (CDPKs), which can sense Ca^2+^ and convert developmental and environmental signals into phosphorylation events. Cytosolic Ca^2+^ levels are modulated by biotic and abiotic stress, generating stress-specific calcium signatures [2].

CDPKs comprise four functional domains: an N-terminal variable domain (ND), a serine/threonine kinase domain (STKD), an auto-inhibitory junction domain (AID) and a regulatory calmodulin-like domain (CaM-LD). Many CDPKs have myristoylation or palmitoylation sites within the ND that facilitate membrane association [3]. PEST motifs within the ND, comprising hydrophilic stretches of at least 12 amino acids, target CDPKs for proteolytic degradation and therefore contribute to their rapid turnover [4]. The STKD is highly conserved and contains two lobes, the N-lobe for ATP binding and the C-lobe for auto-phosphorylation. The AID, which may sometimes be part of the CaM-LD [5], contains a pseudo-substrate sequence that can interact with the active site and inhibit kinase activity. The CaM-LD, which contains four EF-hands, can bind four calcium ions through interactions with eight alpha-helices [5].

CDPKs are tightly regulated by phosphorylation [5] [6]. They have numerous functions in plants, including cytoskeletal reorganization, transcriptional regulation, and the signal transduction of hormonal, stress and defense responses [3]. The CDPK gene family is widely distributed in all green plants, suggesting an ancient origin [7] and that it has undergone significant expansion within the plant kingdom [8]. The 34 CDPKs in *A. thaliana* form a monophyletic family divided into four subfamilies [9], but even in this well-characterized species, only a few CDPKs are characterized for their precise functions [10].

The Vitaceae (creepers and vines) evolved ∼60 million years ago (mya), forming the earliest-diverging lineage of rosids, and it is a sister clade to eurosids I and II [11] [12]. The grapevine genome sequence revealed many triplicates of genes and syntenic blocks [13], suggesting that the ancestral hexaploid genome of *V. vinifera* was derived from an allopolyploidization of two ancestral genomes [12]. However, the triplication may also have occurred following the separation of the monocots and eudicots and before the spread of the eurosids [13]. The proposed allopolyploidization of the grapevine genome is supported by the phylogenetic analysis of 391 predicted nucleotide binding site (NBS) gene family members [12]. The complex evolutionary history of grapevine (without alpha and beta whole-genome duplication as shown in *A. thaliana* [14]) provides an opportunity to study the evolution and functional diversification of ancient duplication events. Furthermore, only one CDPK in grapevine (VvCPK7) has thus far been shown to cause abscisic acid (ABA) hypersensitivity during seed germination, post-germination growth, and stomatal movement [15], but its precise molecular function remains unknown.

## Results and Discussion

### 1. The grapevine genome encodes 17 CDPK genes in four subfamilies

**Figure 1:**
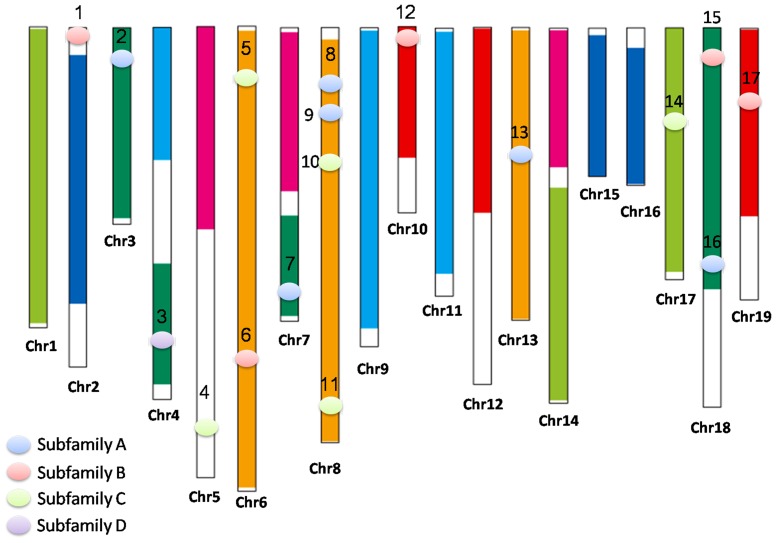
Distribution of CDPK genes on *V. vinifera* chromosomes. Dots in different colors of CDPKs stand for different CDPK groups. Paralogous regions from the putative ancestral grape chromosomes are depicted in the same color from Jaillón et al. (2007) [13].

The grapevine genome contains 17 CDPK genes, all containing the distinct canonical domains (i.e., the STKD and the CaM-LD containing four EF-hands). Besides, 7 CDPK-related kinases (CRKs) were also discovered (VIT_03s0063g00940, VIT_08s0040g01250, VIT_13s0047g00260, VIT_15s0048g02840, VIT_16s0098g01140, VIT_00s0379g00070, VIT_00s0577g00010). The CRKs possess the calmodulin like domain with poor conserved EF hands [16]. The detailed characteristics of the 17 CDPKs are shown (Table 1). The 17 CDPK genes, mapped onto 12 chromosomes (Figure 1), contain well-characterized paralogous regions [13]. We named them as VvCPK1–17 according to convention, based on gene order along the chromosomes [9]. Each VvCPK protein has a predicted sequence of 453–605 amino acids (Figure S1).

**Table 1:** Characteristics of VvCPKs.

Gene Name	Gene identifier	gene locus	EF hands	Gene length	EST	Myristoylaton motif*	palmitoylation prediction**	N-terminal acylation***
**VvCPK1**	VIT_02s0025g00690	chr2:734,407..743,496	4	9.089kb	Y	Y	Y	N
**VvCPK2**	VIT_03s0038g03960	chr3:2,889,737..2,896,406	4	6.669kb	Y	Y	Y	N
**VvCPK3**	VIT_04s0023g03420	chr4:20,005,369..20,011,421	4	6.052kb	Y	Y	Y	N
**VvCPK4**	VIT_05s0102g00170	chr5:22,037,926..22,041,727	4	3.801kb	Y	Y	Y	N
**VvCPK5**	VIT_06s0004g02300	chr6:2,714,924..2,721,335	4	6.411kb	Y	Y	Y	N
**VvCPK6**	VIT_06s0009g03150	chr6:16,322,877..16,325,979	4	3.102kb	N	Y	Y	N
**VvCPK7**	VIT_07s0130g00130	chr7:20,409,616..20,417,336	4	7.72kb	Y	N	Y	N
**VvCPK8**	VIT_08s0032g00780	chr8:4,329,875..4,341,071	4	11.2kb	N	Y	Y	N
**VvCPK9**	VIT_08s0032g01220	chr8:5,882,877..5,886,604	4	3.727kb	N	N	Y	N
**VvCPK10**	VIT_08s0105g00390	chr8:7,622,410..7,641,888	4	19.48kb	Y	Y	Y	N
**VvCPK11**	VIT_08s0007g08300	chr8:21,643,656..21,648,917	4	5.261kb	Y	Y	Y	N
**VvCPK12**	VIT_10s0116g01800	chr10:1,067,284..1,075,645	4	8.361kb	Y	Y	Y	N
**VvCPK13**	VIT_13s0175g00080	chr13:14,945,268..14,952,140	4	6.872kb	N	Y	Y	N
**VvCPK14**	VIT_17s0000g05520	chr17:6,012,943..6,021,623	4	8.68kb	Y	N	N	Y
**VvCPK15**	VIT_18s0001g00990	chr18:1,691,089..1,697,199	4	6.11kb	N	Y	Y	N
**VvCPK16**	VIT_18s0072g01030	chr18:20,502,838..20,524,650	4	21.81kb	Y	N	N	N
**VvCPK17**	VIT_19s0090g00410	chr19:6,574,181..6,579,103	4	4.922kb	Y	Y	Y	N

* Predicted by PlantsP, URL: http://plantsp.genomics.purdue.edu/myrist.html.

** Predicted by CSS-Palm 3.0 software,threshold: high,URL: httP://csspalm.biocuckoo.org/.

*** Predicted by NetAcet 1.0, URL: http://www.cbs.dtu.dk/services/NetAcet/.

**Figure 2:**
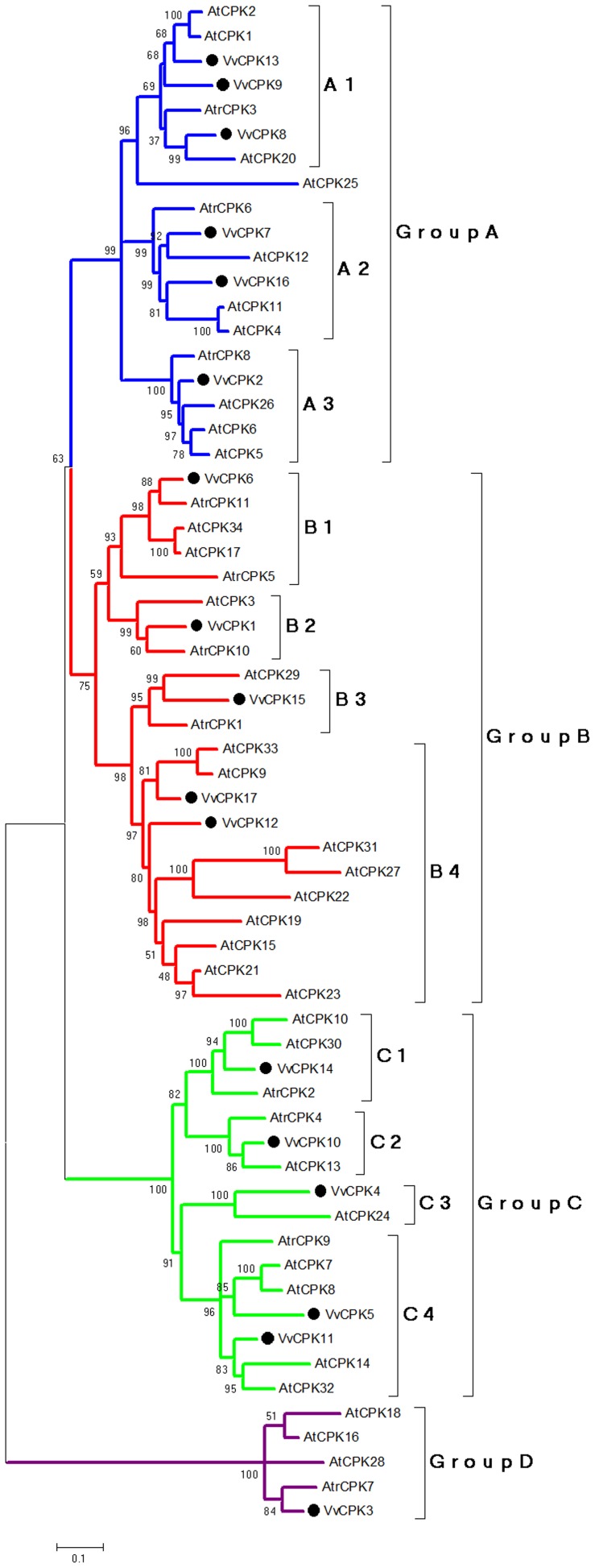
A phylogenetic tree showing the evolutionary relationship of the CDPK family among the *A. thaliana* (At), *V. vinifera* (Vv), and *A. trichopoda*(Atr). Parameters for constructing the Maximum likelihood tree: JTT model, partial deletion, no. of bootstrap replications: 1000.

The phylogenetic relationship among the grapevine CDPK genes was investigated by constructing a maximum-likelihood tree, also containing CDPKs from Amborella (*Amborella trichopoda*; Atr) which is considered the most basal angiosperm, and *Arabidopsis thaliana* (Ath). The 17 VvCPKs were thus assigned to four major subfamilies according to the tree topology (Groups A, B, C, D) matching the classification of the *A. thaliana* CDPKs [9], and further into 12 sub-subfamilies (A1, A2, A3, B1, B2, B3, B4, C1, C2, C3, C4, and D) (Figure 2). We also included the rice (*Oryza sativa*, Os) CDPKs (OsCPKs) in the classification (Figure 2), although OsCPKs have previously been divided into six sub-subfamilies [17].

A careful analysis of the chromosomal distribution of grapevine CDPKs revealed few triple paralogs, in contrast to what was previously shown for the NBS gene family and for the genome as a whole (Figure 1). For example, homologous VvCPKs are present on chromosomes 6 and 8 but not 13, suggesting either that an allopolyploidization event was followed by extensive loss or elimination of sequences from one chromosome.

### 2. The origin and numerical expansion of VvCPK genes

**Figure 3:**
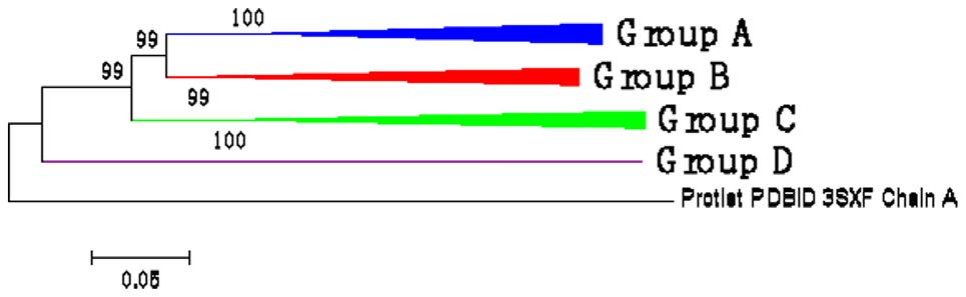
The four subfamilies of VvCPKs form a monophyly (a gene family). The root of the tree was set using the protist (*Toxoplasma Gondii,* Tgo) CDPK as the outgroup.

To determine the root of the four subfamilies, we constructed a tree using the VvCPKs and CDPK 3SXF (PDB ID) from the protist *Toxoplasma gondii*. As shown in the rooted tree (Figure 3), VvCPKs form a monophyletic gene family, suggesting a single origin that radiated into four subfamilies with the subfamily D evolving earliest.

**Figure 4:**
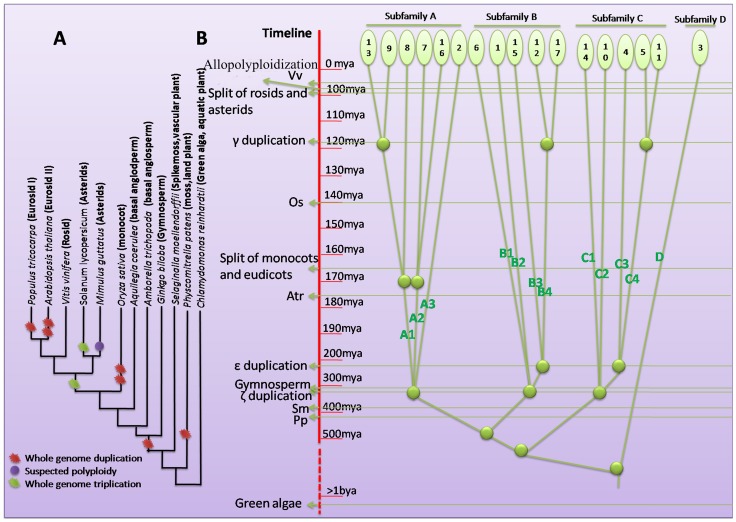
The origin and evolutionary history of the grapevine CDPK gene family. (A) Phylogeny of 12 plant species used to infer the origin and expansions of VvCPKs. The branch lengths are arbitory. (B) Phylogenetic inference tree of the origin and expansions of grapevine CDPK subfamilies. The timeline ranging from 1 bya to present is on the left with important events marked.

To investigate the detailed relationship among the 4 subfamilies and 12 sub-subfamilies, we determined the positions of VvCPKs by phylogenetic inference using CDPKs from other plant species, ranging from green algae to flowering plants (Figure S2). The timeline from 1 billion years ago (bya) to the present day is shown with significant events highlighted, including the emergence of green algae (*Chlamydomonas reinhardtii*), moss (*Physcomitrella patens*, Pp), and the spike moss (*Selaginella moellendorffii*, Sm); the ζ gymnosperm whole-genome duplication resulting in seed plants represented by ginkgo (*Ginkgo biloba,* Gb), the ε duplication resulting in angiosperms represented by *Amborella trichopoda* (Atr), the split between monocots and eudicots represented by rice (*Oryza sativa*, Os), the γ whole-genome duplication which split the rosids and asterids [14] [18], and the putative allopolyploidization which generated the current grapevine genome structure [12] (Figure 4A).

In Figure S3, all green algae CDPKs are shown as outgroup members to all of the four subfamilies, whereas the moss CDPKs are present in all four subfamilies, suggesting that the single origin of the VvCPKs can be dated back to the green algae, before plants colonized the land. This proof supports our hypothesis that the VvCPKs have radiated into four subfamilies before algae and land plants split or in the ancestor of algae and land plants.

According to the tree topology (Figure 3), subfamily D was the earliest date branch and this is supported by sequence comparisons. Two amino acid residues (lysine 189 and glutamic acid 190) were present in the STKD core in sequence alignments (Figure S1). These conserved insertions in subfamily D CDPKs from moss to flowering plants can be used as a marker to differentiate subfamily D from the other three subfamilies (Figure S2).

Because there are few CDPKs in four green algae *Ostreococcus tauri* from the class of *Prasinophyceae*, which is considered as the earliest-diverging green alga [19]), the grapevine CDPKs must have experienced multiple expansion events. As shown in Figure S3, sub-subfamilies A1, A2, A3 include an Amborella CDPK ortholog, whereas subfamilies A2 and A3 include CDPKs from ginkgo (a single-membered clade of seed plants), suggesting that subfamily A predates the origin of seed plants. Likewise, the CDPKs from moss form the outgroups for subfamilies B1, B2, C1, and C2, whereas CDPKs from ginkgo are found in B2 and C2, suggesting that the ζ duplication event in 300–400 mya contributed to B1, B2, C1, and C2 (Figure 4B). The B3 and C4 groups each include a CDPK from the basal angiosperm Amborella, whereas B4 and C3 do not, suggesting that VvCPK15 (B3) is closer to Amborella CDPK1. Because the ginkgo CDPK is an outgroup, the angiosperm ε whole-genome duplication contributed the B3, B4, C3, C4 subfamilies, which appeared before angiosperms but after the seed plant whole-genome duplication. The γ whole-genome duplication, occurred ∼117 mya [18], generated three gene pairs: VvCPK9-VvCPK13, VvCPK12-VvCPK17, and VvCPK5-VvCPK11 (Figure 4B). No expansions have occurred since then, suggesting that all VvCPKs appeared at least 100 mya. The expansion history of the VvCPK gene family does not support a allopolyploidization event approximately 65 mya [12], because no paralogous pairs emerged after the γ duplication. If this allopolyploidization occurred, then all new formed VvCPKs must have been eliminatedby purifying selection.

### 3. Structural divergence among the VvCPKs

**Figure 5:**
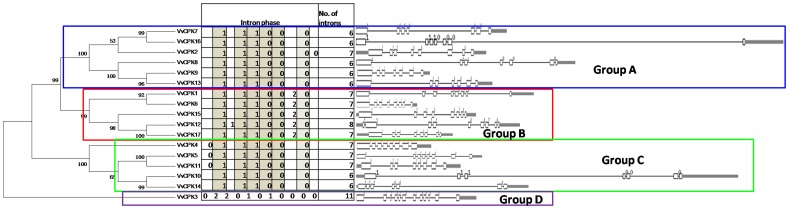
Gene structures of the 17 CDPK genes showing introns and exons. The gene structures were displayed using online tool (http://gsds.cbi.pku.edu.cn/).

We examined the evolutionary differences among the CDPK gene subfamilies by investigating the gene structures of all 17 VvCPK members (Figure 5). The single representative of subfamily D contained 12 exons whereas the other subfamilies contained 7–9 exons. In subfamily A an intron within the EF hand is found between codons, whereas in subfamily B the intron divides codon GGG (glutamic acid) between nucleotides 1402+1403 and 1404, and thus is a phase-2 intron. This insertion is conserved in subfamily B and has not created a frameshift compared to the sequences from subfamily A. Likewise, subfamilies C3 and C4 have phase-0 introns in the ND (Figure 5). These intron insertions, unlikely intron losses in other CDPKs as only C3 and C4 CDPKs have introns in the evolutionary context, are conserved enough to be used as molecular markers to identify families and subfamilies.

**Figure 6:**
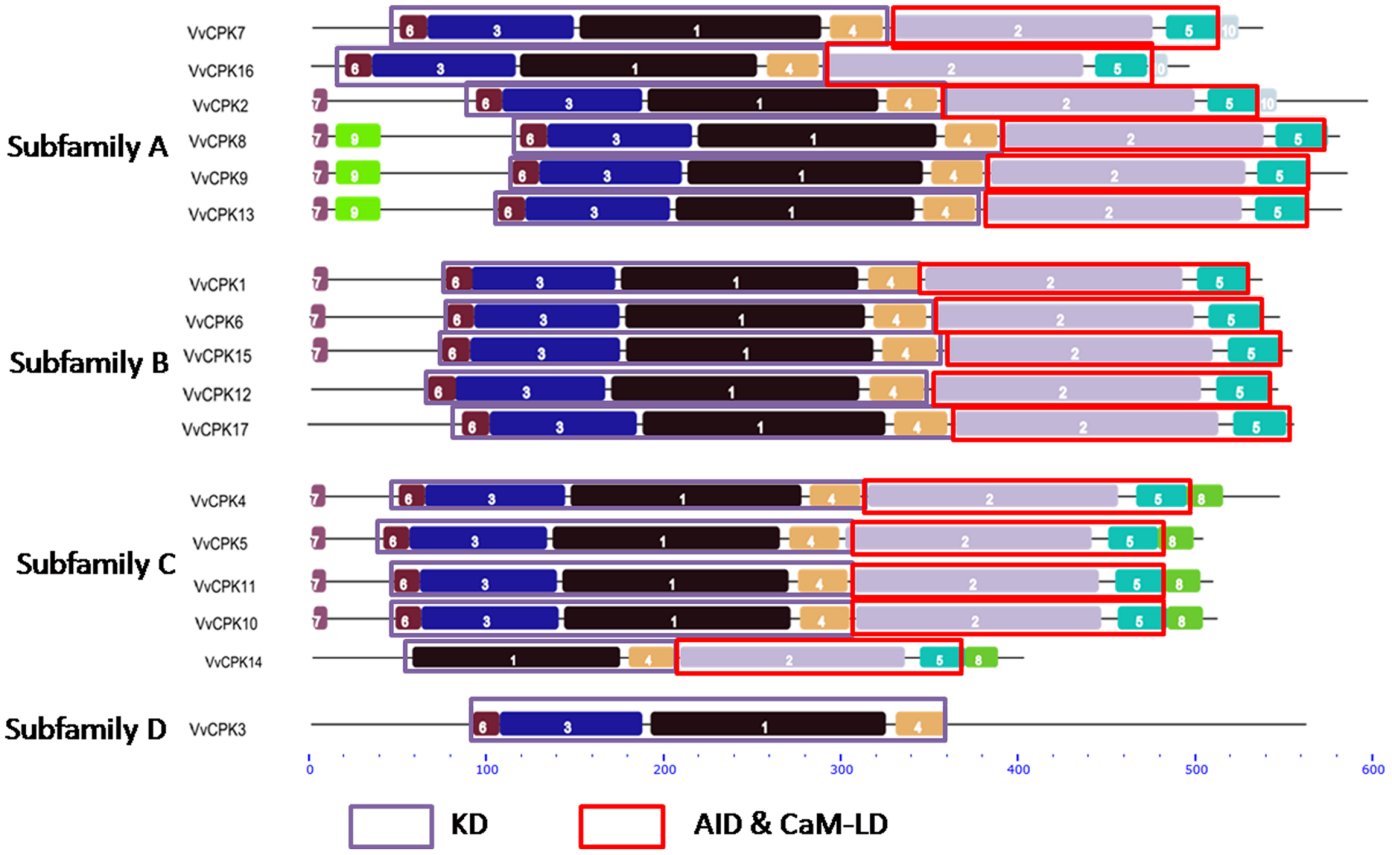
Different motif organizations of 17 VvCPKs. The conserved motifs were detected using MEME online tool (http://meme.sdsc.edu/meme/intro.html).

We identified 10 conserved motifs within the grapevine CDPK genes (Figure 6), which can help to predict their functions [20]. Motifs 6, 3, 1, and 4 constitute the STKD (6 and 3 make up the N-lobe of STKD, whereas the 1 and 4 make up the C-lobe of STKD), whereas the AID and CaM-LD include motifs 2 and 5. We found that the ND was the most variable and that only the ND of sub-subfamily A1 kinases contained motif 9. VvCPKs 3, 7, 12, 14, 16, and 17 possess degenerated or non-conserved NDs, offering indications of functional diversifications. The ND often contains myristoylation or palmitoylation acceptor sites which bind CDPKs to membranes [9]. The variable PEST motifs within the ND help to determine the half-life of the enzyme [4] (Figure S1). VvCPK14 and VvCPK16 appear to possess degenerated N-termini which may contribute to their functional specificity (Figure S1).

**Figure 7:**
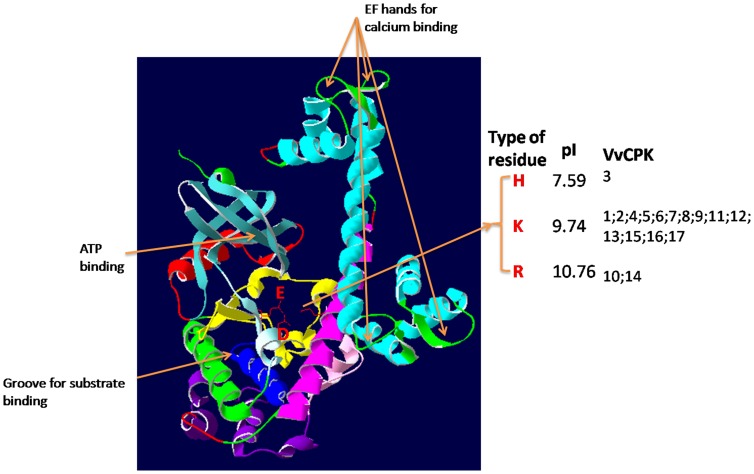
The tertiary structure of the grapevine CDPK. The VvCPK2 is shown as an example. The kinase core is shown in the left with the two residues E and D labeled in red. The auto-inhibitory domain is colored in magenta with the variable residue shown on the right side. The conserved triad with flanking amino acids are shown.

We also modeled the tertiary structure of each VvCPK based on homology with the only *Toxoplasma gondii* CDPK solved to 2.04 Å resolution [5]. This is the first complete model of a plant CDPK since previous structures and models only contained the STKD or CaM-LD [6] [21]. Figure 7 shows the details of the ATP-binding site, the substrate-binding site and the calcium-binding EF hands. The tertiary structure of VvCPK2 reveals the putative mechanism of inhibition and activation mediated by reversible auto-phosphorylation, which involves a putative triad comprising two residues in the STKD and one in the AID [5]. The 17 grapevine CDPKs have putative triads with different confirmations (Figure 7). Most comprise glutamic and aspartic acid residues in the STKD combined with lysine in the AID, but VvCPK3 (subfamily D) has a histidine residue in the AID, and VvCPK10 and 14 have an arginine residue in the AID. However, in each case the triad comprises two acidic residues in the STKD (glutamic and aspartic acid) and a basic amino acid in the AID, i.e. histidine (pI = 7.59), lysine (pI = 9.74), or arginine (pI = 10.76). The efficiency of auto-phosphorylation, therefore, may depend on the interaction between the STKD and AID, which differs among the 17 VvCPKs. For example, VvCPK3 is likely to undergo efficient auto-phosphorylation whereas the process might be more difficult for other VvCPK subfamilies because of the different residues with different pI values in the AID.

### 4. CDPK expression profiles suggest functional divergence

#### 4.1 Expression in the whole plant during development

**Figure 8:**
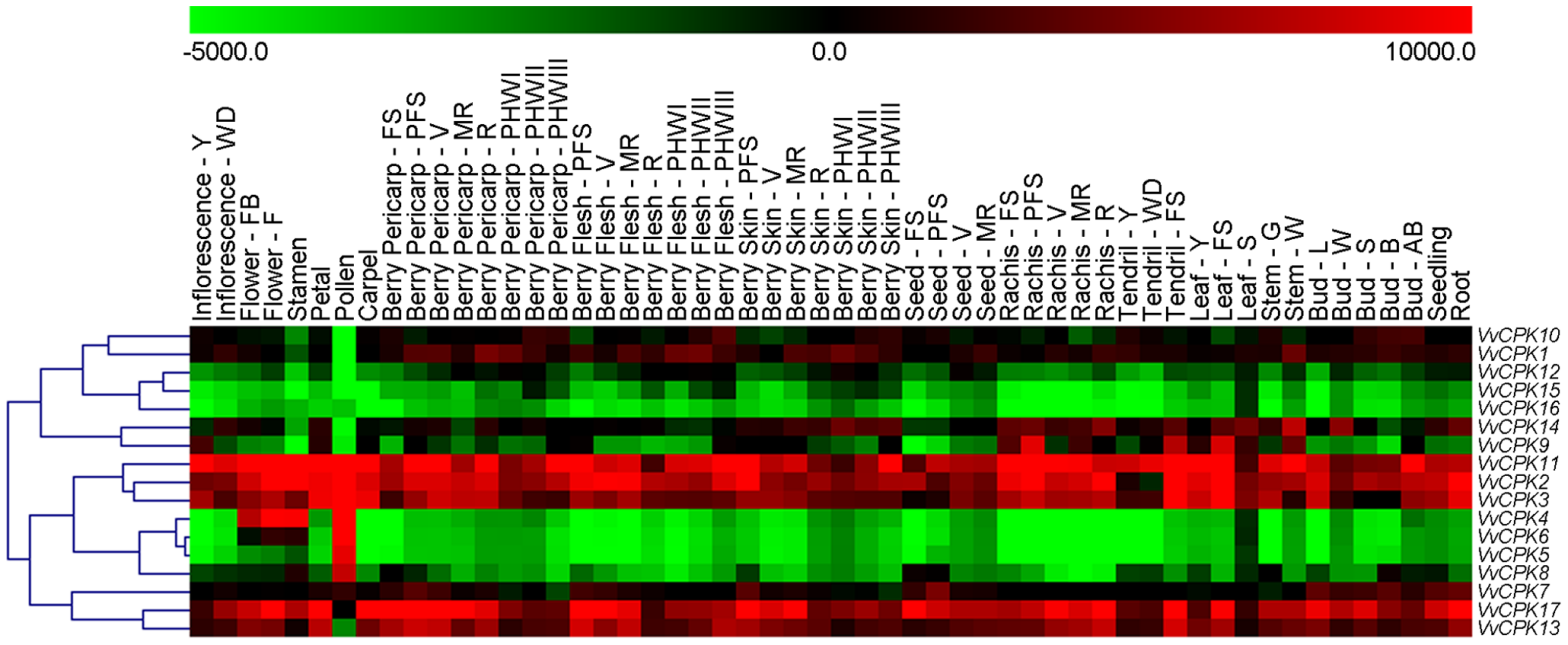
Hierarchical clustering analyses showing the expression of the grape CPDK gene family in the *V. vinifera* cv Corvina atlas. Expression data were normalized based on the mean expression value of each gene in all tissues/organs analyzed. The color scheme used to represent expression level is red/green: black boxes indicate a low variation in expression, green boxes indicate a fold decrease and red boxes indicate a fold increase respect to the mean value of a given gene. Genes were hierarchically clustered based on average Pearson's distance.

We investigated the link between evolutionary and functional divergence by looking at the expression profiles of the 17 CDPK genes in the 54 tissues and developmental stages included in the microarray expression atlas recently reported by Fasoli et al. [22]. Four VvCPKs (4, 5, 6, and 8) were strongly expressed in the pollen. Moreover, VvCPK4 and VvCPK6 were expressed also in stamen and in early stages of flower development, while VvCPK8 expression was detected also in other vegetative and reproductive organs.VvCPK5 was almost exclusively expressed in the pollen and the stamen (Figure 8). Expression pattern of these four VvCPKs were confirmed by real time RT-PCR on pollen, stamen, inflorescence and seed (Figure S4). It is worth to note that VvCPK4 and VvCPK6 were found to be flower and stamen molecular biomarkers in the grapevine gene expression atlas of Fasoli et al. [22]The four VvCPKs highly expressed in pollen were assigned to subfamilies A1, B1, C3, and C4 (Figure 4). Pollen-related CDPK were already described in other species like the *Petunia inflata* CDPK1 and CDPK2, involved in pollen tube growth [23].

Another group of VvCPKs, such as VvCPKs 2, 3, and 11 were expressed in all tissues and developmental stages, suggesting that they perform constitutive housekeeping functions. Interestingly, other VvCPKs were highly expressed in many organs and to an apparent lower extent in pollen (VvCPK13, 17), or in pollen and stamen (VvCPK1, 10, and 14).

The pollen-specific CPKs may be targets for manipulation for breeding self-incompatible lines and seedless cultivars. Pollen development can be prevented and become inviable by disrupting calcium homeostasis, because calcium regulates germination and pollen tube growth [2]. CDPKs are calcium sensors and responders involved in calcium signaling and homeostasis, some CDPK isoforms play distinct roles in pollen tube growth and some play critical functions whereas others do not [23]. Therefore, targeted silencing of pollen-related VvCPK genes (i.e., VvCPKs 4, 5, and 6) may be a potential approach for grapevine breeding, such as producing seedless grapevine, or male and female sterile materials for more efficient breeding.

#### 4.2 VvCPKs are involved in abiotic stress

**Figure 9:**
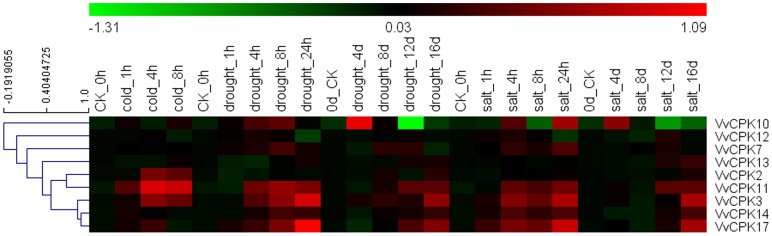
Hierarchical clustering for the expression value under the cold, drought and salt stress. Numbers generated log_2_-fold changes across all the probes.

Grapevine cultivation is sensitive to abiotic stress, particularly extreme temperatures, salinity, and drought [24]. The detailed examination of publicly available Affymetrix microarray data (series matrix accession numbers in NCBI/GEO datasets: GSE31677 and GSE31594) indicated that nine VvCPKs are regulated by different forms of stress, as shown in Figure 9. None of the nine VvCPKs expressed significantly when treated with cold stress, suggesting they are not engaged in the cold stress signaling.VvCPK17 was significantly up-regulated by drought stress in 24 h but not in the long time scale (16d), which is opposite to the expression pattern of VvCPK13. VvCPK3 was up-regulated both in the short and long time scale., Finally, VvCPK7 and 17 were up-regulated under short term salt stress but not long term, in contrast to the expression pattern of VvCPK11, 12, and 14. VvCPK3 was up-regulated both in the short and long time scale under salt stress. All the statistical analyses for each treatment can be seen in the Figure S3. This analysis also indicates that some of the genes that were highlighted as housekeeping from the atlas survey (VvCPK3 and 11) are indeed involved in stress response (Figures 8 and 9).

**Figure 10:**
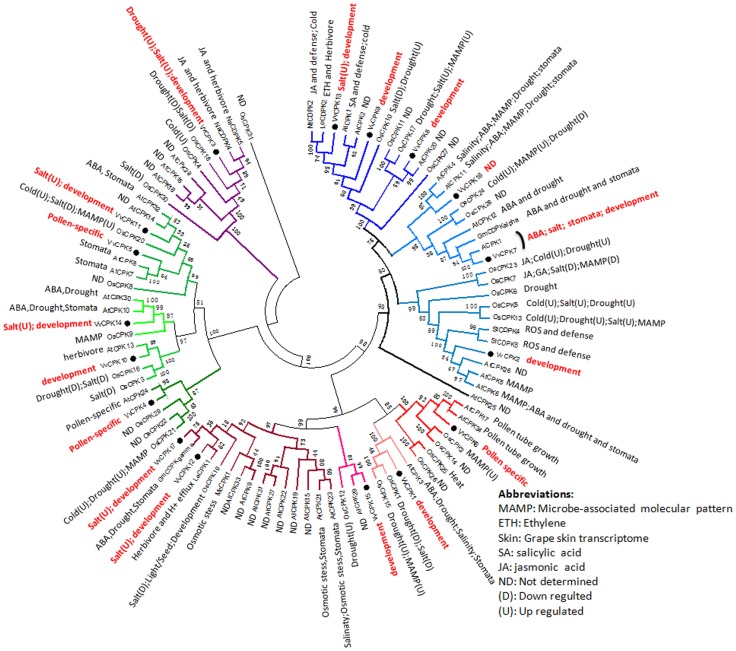
CDPKs from various species involved in cold, drought and salt stress. Genes are clustered into phylogeny tree with functions marked. Functions of the rice CDPKs and arabidopsis CDPKs are from literature while functions of grape CDPKs are from this study.

We compared our phylogenetic and expression data for the VvCPKs with functionally-characterized CDPKs from other species by constructing a phylogenetic tree marked for reported functions (Figure 10) [10] [25]. Almost all of the VvCPKs were responsive to abiotic stress, suggesting that they may be involved in a complex network of cross-talking pathways. However, the pollen-related VvCPKs 4, 5, and 6 have orthologs in *A. thaliana* predominantly representing the C3, C4, and B1 sub-subfamilies, respectively, suggesting either multiple origins or subfunctionalization. Therefore detailed functional characterization is required to determine the specific function of each subfamily and each individual gene, as well as their roles in cross talks in stress responses.

### 5. The fate of VvCPKs after duplication: subfunctionalization and neofunctionalization

Gene duplication and divergence is an important process in the evolution of novel functions [26] [27]. Neofunctionalization is an adaptive process during which one copy of a duplicated gene mutates to adopt a novel function, which cannot be performed by the ancestral sequence. This is one of the mechanisms that can lead to the retention of both copies over long periods of time. Subfunctionalization is a related process in which both duplicated copies diverge to perform more specified functions compared to the ancestral gene, and this can help to explain the short-term fate of the duplicates and can be regarded as a transition state towards neofunctionalization [28]. To investigate the functional diversification of the VvCPKs after duplication, we focused on the paralogs listed in Table 2. The Ka/Ks values for each pair of duplicates were small (all near or below 0.1), and because these pairs were derived from the γ duplication event ∼117 mya, it is likely all these pairs have been under purifying selection since the duplication. The expression pattern of the duplicates is shown in Figure S5. In the gene pairs VvCPK5-VvCPK11, one of the duplicates has become more specific to pollen whereas the other has retained its housekeeping role, suggesting neofunctionalization. However, subfunctionalization was detected in VvCPK8 and VvCPK9, which are expressed in fewer organs/tissues compared to VvCPK13, which might have been closer to the ancestral housekeeping function. The same situation applies to the gene pair VvCPK12-VvCPK17 (Table 2). The comparison of the expression pattern of duplicate genes was verified by real time RT-PCR for the pairs VvCPK5-VvCPK11 and VvCPK8-VvCPK13 (Figure S6).

**Table 2:** Comparison of the duplicates.

Paralog	Myristoylation motif	PEST Motif	Ka	Ks	Ka/Ks	Expression organ	Function
VvCPK5	1	0	0.1259	1.2127	0.1038	stamen and pollen related	pollen development
VvCPK11	1	0				all tissues and organs	Salt
VvCPK9	0	0	0.1135	1.9825	0.0573	Function partitioned	ND
VvCPK13	1	1				all except pollen	drought
VvCPK12	1	0	0.1025	2.2163	0.0463	All except pollen	Salt;
VvCPK17	1	0				all tissues and organs	Drought;Salt
VvCPK8	1	2	0.1775	2.6563	0.0668	Function partitioned	pollen development
VvCPK9	0					Function partitioned	ND
VvCPK8	1	2	0.1378	1.8102	0.0761	Function partitioned	pollen development
VvCPK13	1					all except pollen	drought
VvCPK7	0	0	0.1129	1.469	0.0768	all tissues and organs	ABA and Drought and Stomata
VvCPK16	0	0				Function partitioned	ND

The triplet VvCPK8-VvCPK9-VvCPK13 was resulted from two duplication events, and is characterized by small deviations at the N-terminus (Figure S1). VvCPK9 lacks the myristoylation site and PEST motifs, whereas VvCPK13 has both modifications and VvCPK8 has one myristoylation site as well as two PEST motifs. The myristoylation site is a marker for membrane localization whereas the PEST motifs affect the half-life of the protein, suggesting these deviations have a significant impact on biological functions [4].

The generation of genes with novel functions is often related to the emergence of novel developmental processes. The evolution of pollen-specific genes may be associated with the emergence of seed plants. In the gene pair VvCPK6-VvCPK1, the former is pollen-related whereas the latter is expressed in all tissues except pollen. This gene pair emerged as a result of the ζ whole-genome duplication, which correlated with the landmark evolution of seed plants. The transition required one duplicate to adopt roles in a new physiological process, in this case, pollen development. The gene pair VvCPK10-VvCPk14 was also created by the ζ whole-genome duplication, but in this case both genes have the same expression profile (all tissues and developmental stages except pollen). VvCPK3 evolved along with algae, but is also expressed strongly in the stamen and pollen, suggesting multiple functions with unclear mechanisms.

## Conclusion

### Evolution of the grapevine CDPK gene family

The grapevine genome contains 17 VvCPK genes which are divided into four subfamilies, as previously reported in *A. thaliana*, rice, and as we also showed in moss (*Physcomitrella patens*) and the spike moss (*Selaginella moellendorffii*) by a phylogenetic analysis (Figure S3). The extant VvCPKs resulted from 13 expansions during the last 1 billion years based on the division of the four subfamilies into 12 sub-subfamilies according to the tree topology and gene structure, which will help to investigate expansions in other species including model organisms. Our data revealed the evolutionary origin and expansion of VvCPKs, which is a useful tool for the naming system because the current system is arbitrary and not based on phylogenetic relations among the subfamilies. We therefore suggest that this gene family is, in the future, divided according to its origin and evolutionary history. Thus, subfamilies D, C, A, B in previous reports should be reclassified as subfamilies A, B, C, D, respectively, according to our revised system.

The modeled tertiary structure of the VvCPKs will help to predict their functions as dynamic molecular switches, and the identification of mutations in key residues within the auto-inhibitory triad may provide insight into the different inter-domain binding affinities that regulate CDPK activity. However, the definite regulation by autoinhibitory domain will need to be experimentally determined by approaches like directed mutagenesis of the key amino acids in the autoinhibitory domain.

We discovered two duplicated genes (VvCPK5 and VvCPK8) that gained a peculiar expression in pollen and stamen suggesting neofunctionalization or subfunctionalization, and inferred from the phylogenetic tree that they originated in the same ancestor in seed plants. We also found expression profiles representing neofunctionalization and subfunctionalization in CDPK gene pairs after duplication events. However, the evolution of housekeeping CDPKs into function-partitioned and tissue specific CDPKs requires further investigation.

## Materials and Methods

### Data sources and homology searches

CDPK genes and predicted proteins from different organisms (Table S1) were retrieved and listed as shown in Figure S2. If two or more protein sequences at the same gene locus overlapped, only the longest sequence was used. The core serine/threonine protein kinase domain (STKD, PF00069) was downloaded from Pfam (http://pfam.sanger.ac.uk/). We used the hidden Markov model-based program HMMER v3.0 [29] to search for all proteins containing these two domains. We also used these domains as a BLAST query against the 12x grapevine genome to ensure that all CDPK genes were included. The *Amborella trichopoda*, *Arabidopsis thaliana* and grapevine CDPK uses are shown in Figure S2.

### Sequence alignment, curation, phylogenetic analysis and the calculation of Ka and Ks values

Multiple CDPK sequences were aligned using Muscle with default parameters [30]. Because the predicted VvCPK 7, 14, 16 sequences did not contain the complete N-terminus, we searched for corresponding expressed sequence tags (ESTs) and manually added N-terminal sequences to the VvCPK7 and VvCPK14 (but not to VvCPK16) based on homology comparisons and ESTs FC064179.1 and BM437749.1, respectively. A neighbor-joining consensus tree was generated using MEGA5 [31]. The origin and expansion history of VvCPKs was investigated at high resolution by constructing phylogenetic trees using CDPKs from multiple species. Sequences with conserved domains were screened against the SMART database (www.smart.embl-heidelberg.de) and the conserved domain database (www.ncbi.nlm.nih.gov/Structure/cdd/wrpsb.cgi). We calculated the Ka and Ks values using the YN method [32].

### Homology modeling

Gapped Blast was used to search the SWISS-MODEL template library, and we identified *Toxoplasma gondii* 3SXF chain A at the 2.04 Å resolution in the Protein Data Bank as the optimal template. The e-values for 3SXF and the 17 VvCPKs were approximately 1×10^−60^. We modeled each VvCPK using the SWISS-MODEL program online, followed by evaluation with ERRAT v2.0 (http://nihserver.mbi.ucla.edu/ERRATv2/).

### Expression profiling

The expression profiles of the CDPK gene family were determined using 54 *Vitis vinifera* cv. Corvina (clone 48) samples covering many tissues and developmental stages ([22]): *in vitro* roots, green stem, buds after bud burst (rosette of leaf tips visible), young leaves (leaves collected from shoots with only 5 leaves), senescing leaves (leaves at the beginning of leaf-fall), berry rachis (from fruit-set to ripening), flowers (50% cap-fall) and berry pericarp (from fruit-set to ripe). A set of expression data obtained from berries which had undergone post harvesting withering (for 1 to three months after harvest) was also included (Table S2). The gene expression microarray data were obtained by hybridizations to a NimbleGen microarray 090818_*Vitus_*exp_HX12 (Roche, NimbleGen), which contains probes targeted to 29,549 predicted grapevine genes (http://ddlab.sci.univr.it/FunctionalGenomics/), representing ∼98.6% of the genes predicted from the V1 annotation of the 12x grapevine genome (http://srs.ebi.ac.uk/) and 19,091 random probes as negative controls. The microarray was downloaded from Gene Expression Omnibus (GEO) using the accession number GSE36128.

The expression data (Table S3) were analyzed using T-MeV 4.8.1 (http://www.tm4.org/) software. Data were normalized based on the mean center genes/rows adjustment and Pearson's correlation metric was chosen as statistical metric. Abbreviations after organ names indicate the developmental stage, as indicated in Fasoli et al. (2012) [32]. Expression under abiotic stress was based on the microarray data (series matrix accession numbers GSE31594 and GSE31677) and expression under downloaded from the Gene Expression Omnibus (GEO) databases. Expressional data consist of three replicated treatments and controls, which were first calculated as 2-log-based values and were divided by the control. (Table S3). Statistical analysis of the t-test was performed with software SPSS 13.0.

### RNA extraction, cDNA synthesis and real time RT-PCR

Total RNA was isolated using the Spectrum™ Total RNA kit (SIGMA-Aldrich, St. Louis, MO), with sample-specific modifications of the extraction protocol, as previously described [22]. All RNA samples were firstly treated with DNase I (Promega). First strand cDNA was synthesized using 1 µg of total RNA as the template, obtained by pooling the three biological replicates from the gene expression atlas and the Improm-II™ Reverse Transcription system (Promega), according to the manufacturer's instructions. Gene-specific primers were targeted, when possible, to the 3′-UTR, using ubiquitin (VIT_16s0098g01190) as the reference gene (Table S4). Primers and cDNA were mixed with the Power SYBR® Green PCR Master Mix (Applied Biosystems, Foster City, CA, USA) and the reaction was carried out on a Stratagene MX 3000 P™ QPCR System (Agilent, Technologies, CA) using the following cycling conditions: 95°C hold for 10 min followed by 45 cycles at 95°C for 30 s, 55°C for 30 s and 72°C for 20 s. Nonspecific PCR products were identified by inspecting dissociation curves. Amplification efficiency was calculated from raw data using LingRegPCR software [33]. Standard error (SE) values were calculated as described in Pfaffl MW (2001) [34]. When the Mean Normalized Expression (MNE) value was small (e.g., for *VvCPK4* and *VvCPK8*), the significance of the differential expression between samples (e.g., Pollen and Seed FS) was verified performing a T-test with p<0.01.

## Supporting Information

Figure S1 **Sequence alignment of VvCPKs.** (A): The ND of the CDPKs was highlighted in black box. The PEST motifs and myristoylation motifs are underlined. (B): The black box indicates the kinase core while the red box shows the interacting auto-inhibitory triad. (C): The red box indicates the residue during which the Lys as the key residue in the auto-inhibitory mode.
(TIF)

Figure S2 **Protein Sequences of CDPKs from multiple species ranging from green algae to flowering plants.** Sequences are in fasta format.
(TXT)

Figure S3 **NJ tree for CDPKs from multiple species.**
(TIF)

Figure S4 **Real time RT-PCR on pollen-related VvCPKs.** Transcripts were normalized to the expression of ubiquitin (*UBQ*). Bars indicate standard error (SE) in three technical replicates. The y-axis MNE stands for mean normalized expression. When the MNE (MNE) value was small, the significance of the differential expression between samples was statistically verified (p<0.01).
(TIF)

Figure S5 **Expression pattern of the gene pairs.**
(XLSX)

Figure S6 **Real time RT-PCR of gene pairs.** Transcripts were normalized to the expression of ubiquitin (*UBQ*). Bars indicate standard error (SE) in three technical replicates.
(TIF)

Table S1 **Species used to infer the origin and expansions of VvCPKs.**
(XLSX)

Table S2 **Expression data matrix of VvCPKs, including the expressions in different tissues and organs and under different forms of environmental stress.**
(XLSX)

Table S3 **Data matrix showing the expression value of the VvCPKs.**
(XLSX)

Table S4 **List of primer pairs used in real time RT-PCR analyses.**
(DOCX)
