# Supplementary figures and images for: The Evolutionary History and Diverse Physiological Roles of the Grapevine Calcium-Dependent Protein Kinase Gene Family
Source: PLoS One. 2013 Dec 6;8(12):e80818. doi: 10.1371/journal.pone.0080818 (PMC3855637; doi:10.1371/journal.pone.0080818)

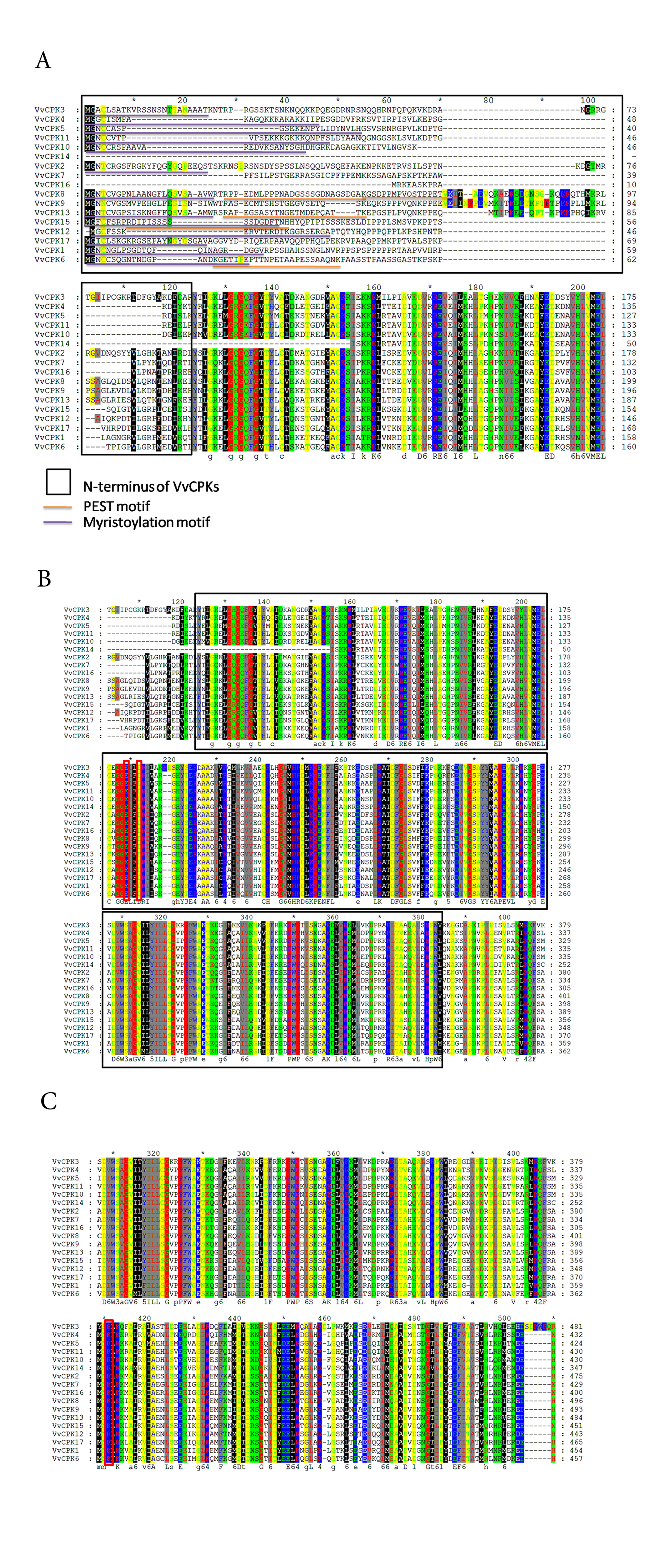

Supplement: Figure S1 — Sequence alignment of VvCPKs. (A): The ND of the CDPKs was highlighted in black box. The PEST motifs and myristoylation motifs are underlined. (B): The black box indicates the kinase core while the red box shows the interacting auto-inhibitory triad. (C): The red box indicates the residue during which the Lys as the key residue in the auto-inhibitory mode. (TIF) [file pone.0080818.s001.tif]

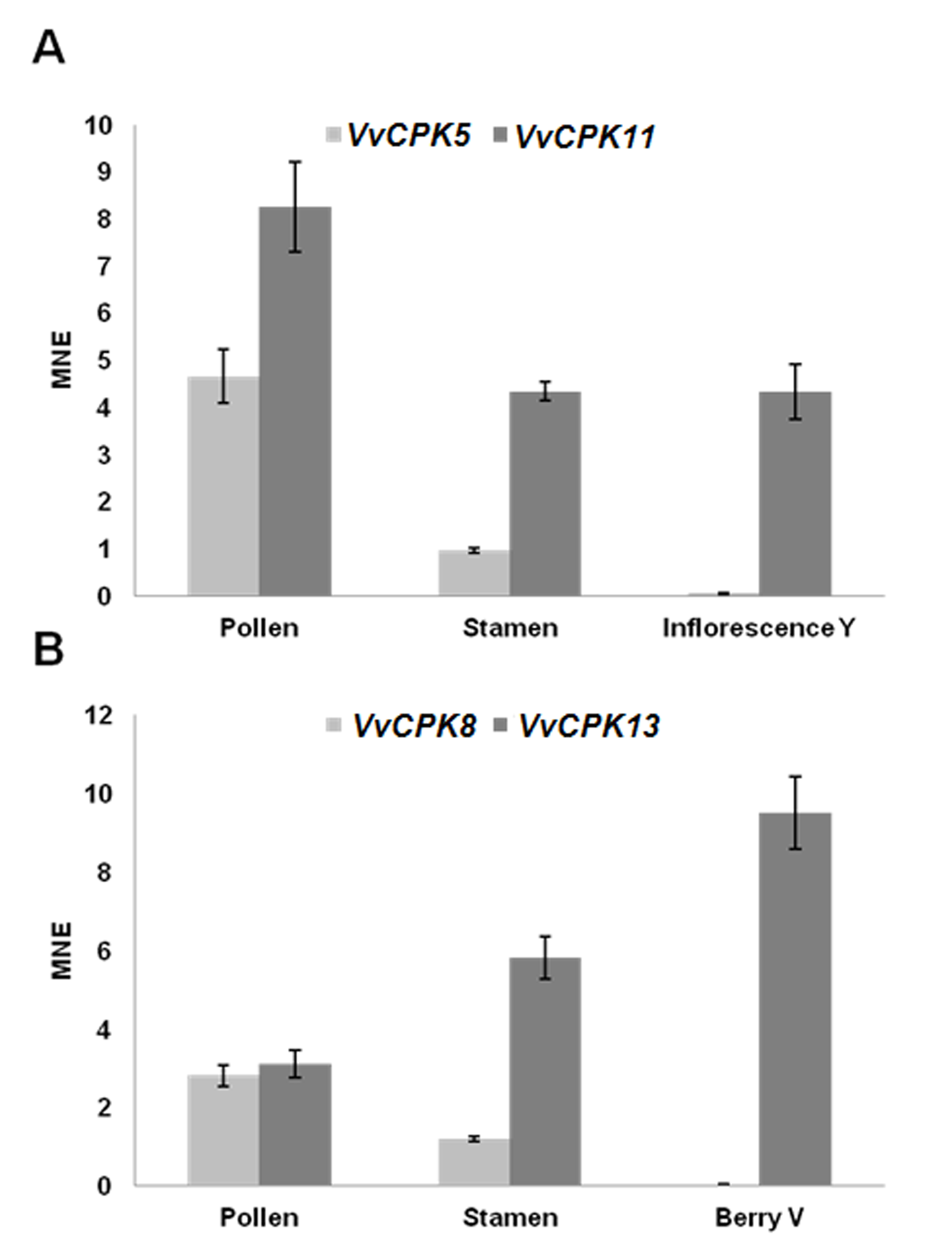

Supplement: Figure S6 — Real time RT-PCR of gene pairs. Transcripts were normalized to the expression of ubiquitin (UBQ). Bars indicate standard error (SE) in three technical replicates. (TIF) [file pone.0080818.s006.tif]
