# Supplementary material for: The Evolutionary History and Diverse Physiological Roles of the Grapevine Calcium-Dependent Protein Kinase Gene Family
Source: PLoS One. 2013 Dec 6;8(12):e80818. doi: 10.1371/journal.pone.0080818 (PMC3855637; doi:10.1371/journal.pone.0080818)
Supplement: Table S4 — List of primer pairs used in real time RT-PCR analyses. (DOCX) [file pone.0080818.s010.docx]

| Gene ID | PN40024  12X V1 ID | Forward primer (5’ to 3’) | Reverse Primer (5’ to 3’) |
| --- | --- | --- | --- |
| ***VvCDKs*** |  |  |  |
| *VvCPK4* | VIT_05s0102g00170 | ACACCTACCCAACCATTCCA | GTTGTGGTTGCCCTTTCTTG |
| *VvCPK5* | VIT_06s0004g02300 | GGAGTTTGCTGCAATGATGA | CTTCCCATCCCTCGTCAACT |
| *VvCPK6* | VIT_06s0009g03150 | AACCGAAGAGTTGGAGCAAG | TCGTAGTTGATCCGGCCATC |
| *VvCPK8* | VIT_08s0032g00780 | GTGAGTTTGTGGCTGCAATG | TGGAGCTCATCTTGGGTGAT |
| *VvCPK11* | VIT_08s0007g08300 | GAACCTCTGGGAGAGGGTTTA | TGCTTTTTCCTTCTCCAACC |
| *VvCPK13* | VIT_13s0175g00080 | TGTTCATTTTCACTTTTCATTTTGA | TCAAGGAAGAGTTGGGTTGC |
| ***UBIQUITIN*** |  |  |  |
| *VvUBQA-52* | VIT_16s0098g01190 | aggcgtgcataacatttgcg | TCTGAGGCTTCGTGGTGGTA |
